# Supplementary material for: A comparative study on fatty acid profile in selected vessels of coronary artery bypass graft (CABG)
Source: PLoS One. 2022 Jan 21;17(1):e0260780. doi: 10.1371/journal.pone.0260780 (PMC8782383; doi:10.1371/journal.pone.0260780)
Supplement: S1 Table — (DOCX) [file pone.0260780.s001.docx]

**S1 Table. Percentages fatty acids in LIMA**

|  |  | LIMA 19 | LIMA 12 | LIMA 26 | LIMA 27 | LIMA 6 | LIMA 7 | LIMA 11 | LIMA 10 | LIMA 13 | LIMA 14 | LIMA 24 | LIMA 9 | Average | SD |
| --- | --- | --- | --- | --- | --- | --- | --- | --- | --- | --- | --- | --- | --- | --- | --- |
| 1 | Dodecanoic acid (n-0) - 12:0 | 14.80519 | 2.74784 | 4.028482 | 9.046868 | 6.253708 | 4.234933 | 5.42819 | 4.463394 | 5.118087 | 7.358633 | 4.245022 | 10.1726 | 6.491912 | 3.402627 |
| 2 | Tetradecanoic acid (n-0) - 14:0 | 17.7779 | 4.867028 | 9.537447 | 10.2587 | 0.370868 | 7.511714 | 7.9781 | 7.839154 | 8.33669 | 13.93625 | 6.759419 | 14.99053 | 9.180316 | 4.674864 |
| 3 | Hexdecanoic acid (n-0) - 16:0 | x | 53.58262 | 3.847239 | 28.92832 | 36.78379 | 35.52881 | 31.84898 | 37.10121 | 38.42108 | 56.32197 | 35.12253 | 39.0003 | 36.04426 | 13.56772 |
| 4 | Hexadecanoic acid (n-1) - 16:1 | 14.4097 | x | 0.127419 | 6.920755 | 2.694779 | 3.004846 | 8.701668 | 5.520826 | 4.583938 | 2.741758 | 4.824476 | 19.7484 | 6.661688 | 5.759235 |
| 5 | Octadecanoic acid (n-0) - 18:0 | 19.71584 | 5.918394 | 8.427556 | 8.674974 | 5.039988 | 5.966805 | 4.184142 | 0.481357 | 5.159381 | x | 4.016675 | 3.377635 | 6.451159 | 4.951089 |
| 6 | Octadecanoic acid(n-1) - 18:1 | 16.95534 | 27.91969 | 60.61507 | 30.07449 | 39.30913 | 37.56149 | 35.78673 | 37.54789 | 32.22537 | 19.6414 | 38.83916 | 4.624961 | 31.75839 | 13.95852 |
| 7 | Octadecanoic acid(n-2) - 18:2 | 16.33603 | 4.964427 | 9.176153 | 2.991141 | 7.315268 | 4.890441 | 4.674084 | 5.521956 | 4.575869 | x | 4.565217 | 0.520054 | 5.957331 | 4.075429 |
| 8 | Eicosenoic acid (n-1) - 20:1 | x | x | 1.923315 | 2.434796 | 1.216456 | 0.800429 | 1.125185 | 0.901215 | 1.256188 | x | 1.627495 | 7.215931 | 2.055668 | 2.002827 |
| 9 | Eicosenoic acid (n-4) - 20:4 | x | x | 0.79114 | 0.669957 | 0.33525 | 0.275936 | 0.27292 | 0.245133 | 0.3234 | x | x | 0.349585 | 0.407915 | 0.204762 |
| 10 | Docosenoic acid(n-4)-22:4 | x | x | 0.714293 | x | 0.255807 | 0.087767 | x | 0.116318 | x | x | x | x | 0.293546 | 0.289947 |
| 11 | Docosenoic acid(n-6)-22:6 | x | x | 0.81189 | x | 0.424962 | 0.136827 | x | 0.261553 | x | x | x | x | 0.408808 | 0.293481 |
