# Supplementary material for: A comparative study on fatty acid profile in selected vessels of coronary artery bypass graft (CABG)
Source: PLoS One. 2022 Jan 21;17(1):e0260780. doi: 10.1371/journal.pone.0260780 (PMC8782383; doi:10.1371/journal.pone.0260780)
Supplement: S2 Table — (DOCX) [file pone.0260780.s002.docx]

**S2 Table. Percentages fatty acids in SV**

|  |  | Vein 28 | Vein 23 | Vein 20 | Vein 22 | LSV 16 | LSV 18 | LSV 4 | Vein 7 | LSV 9 | Vein 27 | Vein 8 | Vein 15 | LSV 14 | Vein 19 | Vein 6 | Vein 10 | LSV 11 | Vein 25 | Vein 21 | Average | SD |
| --- | --- | --- | --- | --- | --- | --- | --- | --- | --- | --- | --- | --- | --- | --- | --- | --- | --- | --- | --- | --- | --- | --- |
| 1 | Dodecanoic acid (n-0) - 12:0 | 1.896155 | 17.18487 | x | x | x | 2.952412 | 2.55524 | 0.682508 | 2.575866 | 0.433817 | 2.084015 | 4.497695 | 1.393446 | 2.51653 | 2.756103 | x | 2.182666 | x | 4.082034 | 3.413811 | 4.11529 |
| 2 | Tetradecanoic acid (n-0) - 14:0 | 2.559276 | 46.8901 | x | 8.958218 | 4.23076 | 5.16413 | 6.093423 | 2.122231 | 5.143763 | 0.86414 | 6.35873 | 8.221633 | 3.738176 | 4.245626 | 5.896622 | 2.126802 | 4.535544 | 4.32193 | 7.251602 | 7.151261 | 10.14459 |
| 3 | Hexdecanoic acid (n-0) - 16:0 | 7.470787 | x | x | 34.92439 | 39.64635 | 24.13024 | 32.29181 | 30.97631 | 31.50638 | 7.064394 | 30.41913 | 32.70399 | 38.4309 | 37.67982 | 52.62038 | 18.44249 | 28.02754 | 30.80235 | 40.41214 | 30.44408 | 11.41884 |
| 4 | Hexadecanoic acid (n-1) - 16:1 | 2.790031 | 17.00018 | x | 9.340535 | 2.654591 | 13.11152 | 11.26902 | 2.659363 | 8.386326 | 1.163906 | 11.10832 | 9.176346 | 3.514304 | 9.230322 | x | 4.399804 | 10.31108 | 5.342099 | 7.968472 | 7.613307 | 4.374942 |
| 5 | Octadecanoic acid (n-0) - 18:0 | 1.206995 | 6.930816 | 25.93225 | 3.777702 | 13.10333 | 4.374968 | 4.337733 | 10.1687 | 8.138106 | 0.721896 | 2.580837 | 3.820658 | 8.276183 | 6.115262 | x | 47.03599 | 5.811718 | 15.58507 | 5.281855 | 9.622226 | 11.09739 |
| 6 | Octadecanoic acid(n-1) - 18:1 | 82.79373 | 4.33138 | 74.06775 | 39.78894 | 27.64618 | 36.47576 | 37.08032 | 33.79583 | 35.22958 | 88.40971 | 42.63715 | 32.35434 | 37.3681 | 40.21244 | 34.01392 | 20.46406 | 41.02206 | 22.97651 | 35.0039 | 40.29851 | 20.61081 |
| 7 | Octadecanoic acid(n-2) - 18:2 | 1.283028 | x | x | 1.779308 | 3.678174 | 6.651051 | 6.37246 | 8.443068 | 0.418678 | 0.892827 | 1.864066 | 4.74809 | x | x | 4.712977 | 2.129256 | 5.061835 | 4.879849 | x | 3.779619 | 2.443932 |
| 8 | Eicosenoic acid (n-1) - 20:1 | x | x | x | 1.430905 | x | 1.036722 | x | x | 1.408209 | 0.349901 | 2.363634 | 3.618658 | 7.278897 | x | x | x | x | x | x | 3.779619 | 2.352549 |
| 9 | Eicosenoic acid (n-4) - 20:4 | x | 7.662652 | x | x | 9.040615 | 4.042848 | x | 6.571855 | 4.749951 | 0.07682 | 0.478395 | 0.590541 | x | x | x | 5.401587 | 3.047559 | 13.26231 | x | 4.993194 | 4.040176 |
| 10 | Docosenoic acid(n-3)-22:3 | x | x | x | x | x | x | x | 1.571639 | 0.735396 | 0.022594 | x | x | x | x | x | x | x | x | x | 4.993194 | 0.775342 |
| 11 | Docosenoic acid(n-6)-22:6 | x | x | x | x | x | 2.060347 | x | 3.008491 | 1.707745 | x | 0.105725 | 0.268055 | x | x | x | x | x | 2.829882 | x | 1.663374 | 1.241195 |
