# Supplementary material for: A comparative study on fatty acid profile in selected vessels of coronary artery bypass graft (CABG)
Source: PLoS One. 2022 Jan 21;17(1):e0260780. doi: 10.1371/journal.pone.0260780 (PMC8782383; doi:10.1371/journal.pone.0260780)
Supplement: S3 Table — (DOCX) [file pone.0260780.s003.docx]

**S3 Table. Percentages fatty acids in RA**

|  |  | Radial 26 | Radial 13 | Radial 8 | Radial 19 | Radial 7 | Radial 25 | Radial 5 | Radial 15 | Radial 21 | Average | SD |
| --- | --- | --- | --- | --- | --- | --- | --- | --- | --- | --- | --- | --- |
| 1 | Dodecanoic acid (n-0) - 12:0 | 0.106788 | 4.051757 | 3.310923 | 2.864431 | 6.45036 | 0.754067 | 0.849587 | 4.704119 | 6.77619 | 3.318691 | 2.434516 |
| 2 | Tetradecanoic acid (n-0) - 14:0 | 5.79412 | 7.294916 | 7.518746 | 4.733856 | 6.798062 | 9.998845 | 2.984571 | 8.468065 | 12.39502 | 7.3318 | 2.792261 |
| 3 | Hexdecanoic acid (n-0) -16:0 | 34.06764 | 34.59026 | 40.36333 | 38.08172 | 39.28715 | 36.96898 | 29.02753 | 33.55688 | 0.62611 | 31.84107 | 12.20176 |
| 4 | Hexadecanoic acid (n-1) - 16:1 | 0.643912 | 7.318075 | 2.980646 | 6.964464 | 3.51319 | 7.472089 | 6.441791 | 7.289143 | 5.893463 | 5.390753 | 2.432867 |
| 5 | Octadecanoic acid (n-0) - 18:0 | 4.531675 | 4.340236 | 6.362247 | 8.381852 | 6.088149 | 4.394727 | 4.306201 | 3.539484 | 10.07653 | 5.780122 | 2.19106 |
| 6 | Octadecanoic acid(n-1) - 18:1 | 45.81096 | 41.36757 | 35.60603 | 34.9932 | 29.55651 | 40.41129 | 43.43161 | 36.00383 | 58.17307 | 40.5949 | 8.246733 |
| 7 | Octadecanoic acid(n-2) - 18:2 | 7.082825 | x | 2.433208 | 3.980478 | 4.751173 | x | 11.16923 | 5.082714 | 6.059624 | 5.794179 | 2.792905 |
| 8 | Eicosenoic acid (n-1) - 20:1 | 1.202232 | 1.037186 | 1.424868 | x | 2.151615 | x | 0.839025 | 0.673995 | x | 1.221487 | 0.526662 |
| 9 | Eicosenoic acid (n-4) - 20:4 | 0.422411 | x | x | x | 1.403797 | x | 0.477879 | 0.373684 | x | 0.669443 | 0.491417 |
| 10 | Docosenoic acid(n-3)-22:3 | x | x | x | x | x | x | 0.22446 | 0.097042 | x | 0.160751 | 0.090098 |
| 11 | Docosenoic acid(n-6)-22:6 | 0.337435 | x | x | x | x | x | 0.248119 | 0.21105 | x | 0.265535 | 0.064967 |
