# Supplementary material for: A comparative study on fatty acid profile in selected vessels of coronary artery bypass graft (CABG)
Source: PLoS One. 2022 Jan 21;17(1):e0260780. doi: 10.1371/journal.pone.0260780 (PMC8782383; doi:10.1371/journal.pone.0260780)
Supplement: S4 Table — (DOCX) [file pone.0260780.s004.docx]

**S4 Table. Linear regression Analysis**

**S4 Table A. Linear regression of lauric acid in SV with confounding variables**

| **Coefficients^a^** | | | | | | | | |
| --- | --- | --- | --- | --- | --- | --- | --- | --- |
| Model | | Unstandardized Coefficients | | Standardized Coefficients | t | Sig. | 95.0% Confidence Interval for B | |
|  |  | B | Std. Error | Beta |  |  | Lower Bound | Upper Bound |
| 1 | (Constant) | 8.132 | 15.541 |  | .523 | .612 | -26.495 | 42.759 |
|  | Age | -.086 | .115 | -.237 | -.748 | .471 | -.342 | .170 |
|  | Gender | 2.783 | 3.402 | .344 | .818 | .432 | -4.797 | 10.362 |
|  | HTN | .602 | 2.714 | .081 | .222 | .829 | -5.446 | 6.650 |
|  | HL | -.463 | 2.205 | -.062 | -.210 | .838 | -5.376 | 4.450 |
|  | FH | 2.079 | 1.895 | .336 | 1.097 | .298 | -2.143 | 6.301 |
|  | Smoking | 1.479 | 2.540 | .222 | .582 | .573 | -4.182 | 7.139 |
|  | MI | -1.028 | 3.532 | -.147 | -.291 | .777 | -8.897 | 6.841 |
|  | Cho | -.010 | .052 | -.083 | -.186 | .856 | -.124 | .105 |
|  | LDL | -.020 | .077 | -.139 | -.266 | .796 | -.191 | .150 |
|  | HDL | .041 | .140 | .099 | .290 | .777 | -.272 | .353 |
|  | Tri | -.003 | .015 | -.088 | -.211 | .837 | -.037 | .031 |
|  | DM | -2.562 | 2.607 | -.417 | -.982 | .349 | -8.371 | 3.248 |
| a. Dependent Variable: Lauric acid in SV  Independent variables: HTN – hypertension, HL- hyperlipidemia, FH- Family history of CAD, MI- Prior myocardial infarction, Cho- Cholesterol, LDL- Low density lipoprotein, HDL- High density lipoprotein, Tri- Triglyceride, DM- Diabetic Mellitus | | | | | | | | |

**S4 Table B. Linear regression of lauric acid in LIMA with confounding variables**

| **Coefficients^a^** | | | | | | | | |
| --- | --- | --- | --- | --- | --- | --- | --- | --- |
| Model | | Unstandardized Coefficients | | Standardized Coefficients | t | Sig. | 95.0% Confidence Interval for B | |
|  |  | B | Std. Error | Beta |  |  | Lower Bound | Upper Bound |
| 1 | (Constant) | 13.466 | 11.305 |  | 1.191 | .261 | -11.723 | 38.656 |
|  | Age | .089 | .084 | .298 | 1.062 | .313 | -.097 | .275 |
|  | Gender | 2.101 | 2.474 | .317 | .849 | .416 | -3.413 | 7.614 |
|  | HTN | 2.126 | 1.975 | .349 | 1.077 | .307 | -2.274 | 6.526 |
|  | HL | 2.032 | 1.604 | .333 | 1.267 | .234 | -1.542 | 5.606 |
|  | FH | -2.378 | 1.378 | -.469 | -1.725 | .115 | -5.449 | .693 |
|  | Smoking | -1.832 | 1.848 | -.335 | -.991 | .345 | -5.949 | 2.286 |
|  | MI | -.261 | 2.569 | -.046 | -.102 | .921 | -5.986 | 5.463 |
|  | Cho | -.046 | .037 | -.480 | -1.216 | .252 | -.129 | .038 |
|  | LDL | .001 | .056 | .005 | .011 | .992 | -.124 | .125 |
|  | HDL | -.118 | .102 | -.350 | -1.157 | .274 | -.345 | .109 |
|  | Tri | -.001 | .011 | -.042 | -.113 | .912 | -.026 | .024 |
|  | DM | -1.587 | 1.897 | -.315 | -.837 | .422 | -5.814 | 2.639 |
| a. Dependent Variable: Lauric acid in LIMA  Independent variables: HTN – hypertension, HL- hyperlipidemia, FH- Family history of CAD, MI- Prior myocardial infarction, Cho- Cholesterol, LDL- Low density lipoprotein, HDL- High density lipoprotein, Tri- Triglyceride, DM- Diabetic Mellitus | | | | | | | | |

**S4 Table C. Linear regression of lauric acid in RA with confounding variables**

| **Coefficients^a^** | | | | | | | | |
| --- | --- | --- | --- | --- | --- | --- | --- | --- |
| Model | | Unstandardized Coefficients | | Standardized Coefficients | t | Sig. | 95.0% Confidence Interval for B | |
|  |  | B | Std. Error | Beta |  |  | Lower Bound | Upper Bound |
| 1 | (Constant) | 5.726 | 7.119 |  | .804 | .440 | -10.136 | 21.588 |
|  | Age | -.020 | .053 | -.112 | -.382 | .711 | -.137 | .097 |
|  | Gender | -.713 | 1.558 | -.178 | -.457 | .657 | -4.185 | 2.759 |
|  | HTN | -2.010 | 1.243 | -.546 | -1.616 | .137 | -4.780 | .761 |
|  | HL | .171 | 1.010 | .046 | .169 | .869 | -2.080 | 2.421 |
|  | FH | 1.922 | .868 | .627 | 2.214 | .051 | -.012 | 3.856 |
|  | Smoking | -.277 | 1.164 | -.084 | -.238 | .817 | -2.869 | 2.316 |
|  | MI | -.582 | 1.618 | -.168 | -.360 | .727 | -4.187 | 3.023 |
|  | Cho | -.001 | .024 | -.010 | -.025 | .981 | -.053 | .052 |
|  | LDL | -.040 | .035 | -.550 | -1.136 | .282 | -.118 | .038 |
|  | HDL | .109 | .064 | .536 | 1.699 | .120 | -.034 | .252 |
|  | Tri | -.002 | .007 | -.099 | -.258 | .802 | -.017 | .014 |
|  | DM | .560 | 1.194 | .184 | .469 | .649 | -2.101 | 3.221 |
| a. Dependent Variable: Lauric acid in RA  Independent variables: HTN – hypertension, HL- hyperlipidemia, FH- Family history of CAD, MI- Prior myocardial infarction, Cho- Cholesterol, LDL- Low density lipoprotein, HDL- High density lipoprotein, Tri- Triglyceride, DM- Diabetic Mellitus | | | | | | | | |

**S4 Table D. Linear regression of arachidonic acid acid in SV with confounding variables**

| **Coefficients^a^** | | | | | | | | |
| --- | --- | --- | --- | --- | --- | --- | --- | --- |
| Model | | Unstandardized Coefficients | | Standardized Coefficients | t | Sig. | 95.0% Confidence Interval for B | |
|  |  | B | Std. Error | Beta |  |  | Lower Bound | Upper Bound |
| 1 | (Constant) | 5.808 | 9.381 |  | .619 | .550 | -15.095 | 26.711 |
|  | Age | -.112 | .069 | -.402 | -1.619 | .137 | -.267 | .042 |
|  | Gender | -4.053 | 2.053 | -.651 | -1.974 | .077 | -8.628 | .522 |
|  | HTN | -1.086 | 1.639 | -.190 | -.663 | .522 | -4.737 | 2.565 |
|  | HL | 1.938 | 1.331 | .339 | 1.456 | .176 | -1.028 | 4.904 |
|  | FH | 2.996 | 1.144 | .629 | 2.619 | .026 | .447 | 5.544 |
|  | Smoking | 3.642 | 1.534 | .710 | 2.375 | .039 | .225 | 7.059 |
|  | MI | -.706 | 2.132 | -.131 | -.331 | .747 | -5.456 | 4.044 |
|  | Cho | .011 | .031 | .125 | .358 | .728 | -.058 | .080 |
|  | LDL | -.021 | .046 | -.186 | -.452 | .661 | -.124 | .082 |
|  | HDL | .100 | .085 | .318 | 1.187 | .263 | -.088 | .289 |
|  | Tri | -.010 | .009 | -.350 | -1.074 | .308 | -.030 | .011 |
|  | DM | -.593 | 1.574 | -.126 | -.377 | .714 | -4.101 | 2.914 |
| a. Dependent Variable: Arachidonic acid in SV  Independent variables: HTN – hypertension, HL- hyperlipidemia, FH- Family history of CAD, MI- Prior myocardial infarction, Cho- Cholesterol, LDL- Low density lipoprotein, HDL- High density lipoprotein, Tri- Triglyceride, DM- Diabetic Mellitus | | | | | | | | |

**S4 Table E. Linear regression of arachidonic acid in LIMA with confounding variables**

| **Coefficients^a^** | | | | | | | | |
| --- | --- | --- | --- | --- | --- | --- | --- | --- |
| Model | | Unstandardized Coefficients | | Standardized Coefficients | t | Sig. | 95.0% Confidence Interval for B | |
|  |  | B | Std. Error | Beta |  |  | Lower Bound | Upper Bound |
| 1 | (Constant) | .059 | .812 |  | .073 | .944 | -1.749 | 1.867 |
|  | Age | .003 | .006 | .151 | .542 | .600 | -.010 | .017 |
|  | Gender | .409 | .178 | .850 | 2.300 | .044 | .013 | .804 |
|  | HTN | .204 | .142 | .463 | 1.442 | .180 | -.111 | .520 |
|  | HL | .028 | .115 | .064 | .247 | .810 | -.228 | .285 |
|  | FH | -.136 | .099 | -.370 | -1.373 | .200 | -.356 | .085 |
|  | Smoking | -.237 | .133 | -.598 | -1.786 | .104 | -.533 | .059 |
|  | MI | -.080 | .184 | -.193 | -.434 | .674 | -.491 | .331 |
|  | Cho | .004 | .003 | .533 | 1.364 | .202 | -.002 | .010 |
|  | LDL | -.004 | .004 | -.429 | -.932 | .373 | -.013 | .005 |
|  | HDL | -.009 | .007 | -.359 | -1.198 | .258 | -.025 | .008 |
|  | Tri | .001 | .001 | .235 | .643 | .535 | -.001 | .002 |
|  | DM | .048 | .136 | .131 | .350 | .734 | -.256 | .351 |
| a. Dependent Variable: Arachidonic acid in LIMA  Independent variables: HTN – hypertension, HL- hyperlipidemia, FH- Family history of CAD, MI- Prior myocardial infarction, Cho- Cholesterol, LDL- Low density lipoprotein, HDL- High density lipoprotein, Tri- Triglyceride, DM- Diabetic Mellitus | | | | | | | | |

**S4 Table F. Linear regression of arachidonic acid in RA with confounding variables**

| **Coefficients^a^** | | | | | | | | |
| --- | --- | --- | --- | --- | --- | --- | --- | --- |
| Model | | Unstandardized Coefficients | | Standardized Coefficients | t | Sig. | 95.0% Confidence Interval for B | |
|  |  | B | Std. Error | Beta |  |  | Lower Bound | Upper Bound |
| 1 | (Constant) | 3.971 | 5.778 |  | .687 | .508 | -8.903 | 16.845 |
|  | Age | -.024 | .043 | -.198 | -.557 | .590 | -.119 | .071 |
|  | Gender | -1.570 | 1.265 | -.585 | -1.241 | .243 | -4.388 | 1.248 |
|  | HTN | -1.481 | 1.009 | -.600 | -1.468 | .173 | -3.730 | .767 |
|  | HL | .330 | .820 | .134 | .403 | .695 | -1.496 | 2.157 |
|  | FH | .429 | .704 | .209 | .609 | .556 | -1.141 | 1.999 |
|  | Smoking | 1.369 | .944 | .619 | 1.449 | .178 | -.736 | 3.473 |
|  | MI | -.075 | 1.313 | -.032 | -.057 | .956 | -3.001 | 2.851 |
|  | Cho | .006 | .019 | .158 | .318 | .757 | -.037 | .049 |
|  | LDL | 7.832E-5 | .028 | .002 | .003 | .998 | -.063 | .064 |
|  | HDL | -.005 | .052 | -.039 | -.102 | .921 | -.121 | .111 |
|  | Tri | -.003 | .006 | -.272 | -.585 | .571 | -.016 | .009 |
|  | DM | -.239 | .969 | -.117 | -.246 | .811 | -2.398 | 1.921 |
| a. Dependent Variable: Arachidonic acid in RA  Independent variables: HTN – hypertension, HL- hyperlipidemia, FH- Family history of CAD, MI- Prior myocardial infarction, Cho- Cholesterol, LDL- Low density lipoprotein, HDL- High density lipoprotein, Tri- Triglyceride, DM- Diabetic Mellitus | | | | | | | | |

**S4 Table G. Linear regression of AA, ω-6/ DHA, ω-3 in SV with confounding variables**

| **Coefficients^a^** | | | | | | | | |
| --- | --- | --- | --- | --- | --- | --- | --- | --- |
| Model | | Unstandardized Coefficients | | Standardized Coefficients | t | Sig. | 95.0% Confidence Interval for B | |
|  |  | B | Std. Error | Beta |  |  | Lower Bound | Upper Bound |
| 1 | (Constant) | 10.425 | 6.025 |  | 1.730 | .114 | -3.000 | 23.849 |
|  | Age | -.018 | .045 | -.139 | -.401 | .697 | -.117 | .081 |
|  | Gender | -.338 | 1.319 | -.118 | -.257 | .803 | -3.277 | 2.600 |
|  | HTN | .219 | 1.052 | .083 | .208 | .840 | -2.126 | 2.563 |
|  | HL | 1.013 | .855 | .385 | 1.185 | .263 | -.891 | 2.918 |
|  | FH | -.280 | .735 | -.128 | -.381 | .711 | -1.916 | 1.357 |
|  | Smoking | -.172 | .985 | -.073 | -.175 | .865 | -2.366 | 2.022 |
|  | MI | -1.278 | 1.369 | -.517 | -.934 | .372 | -4.329 | 1.772 |
|  | Cho | -.005 | .020 | -.130 | -.266 | .796 | -.050 | .039 |
|  | LDL | -.007 | .030 | -.142 | -.247 | .810 | -.074 | .059 |
|  | HDL | -.065 | .054 | -.446 | -1.192 | .261 | -.186 | .056 |
|  | Tri | -.004 | .006 | -.325 | -.714 | .492 | -.017 | .009 |
|  | DM | -.738 | 1.011 | -.340 | -.730 | .482 | -2.990 | 1.514 |
| a. Dependent Variable: AA, ω-6/ DHA, ω-3 in SV  Independent variables: HTN – hypertension, HL- hyperlipidemia, FH- Family history of CAD, MI- Prior myocardial infarction, Cho- Cholesterol, LDL- Low density lipoprotein, HDL- High density lipoprotein, Tri- Triglyceride, DM- Diabetic Mellitus | | | | | | | | |

**S4 Table H. Linear regression of AA, ω-6/ DHA, ω-3 in LIMA with confounding variables**

| **Coefficients^a^** | | | | | | | | |
| --- | --- | --- | --- | --- | --- | --- | --- | --- |
| Model | | Unstandardized Coefficients | | Standardized Coefficients | t | Sig. | 95.0% Confidence Interval for B | |
|  |  | B | Std. Error | Beta |  |  | Lower Bound | Upper Bound |
| 1 | (Constant) | .811 | 2.835 |  | .286 | .781 | -5.505 | 7.128 |
|  | Age | -.035 | .021 | -.501 | -1.651 | .130 | -.081 | .012 |
|  | Gender | .229 | .620 | .149 | .370 | .719 | -1.153 | 1.612 |
|  | HTN | -.636 | .495 | -.450 | -1.285 | .228 | -1.740 | .467 |
|  | HL | -.086 | .402 | -.061 | -.213 | .836 | -.982 | .810 |
|  | FH | .198 | .346 | .169 | .574 | .579 | -.572 | .969 |
|  | Smoking | .034 | .463 | .027 | .074 | .942 | -.998 | 1.067 |
|  | MI | .676 | .644 | .509 | 1.049 | .319 | -.760 | 2.111 |
|  | Cho | .004 | .009 | .176 | .413 | .688 | -.017 | .025 |
|  | LDL | .000 | .014 | .011 | .023 | .982 | -.031 | .031 |
|  | HDL | .020 | .026 | .259 | .791 | .447 | -.037 | .077 |
|  | Tri | .001 | .003 | .164 | .411 | .690 | -.005 | .007 |
|  | DM | .697 | .476 | .596 | 1.465 | .174 | -.363 | 1.757 |
| a. Dependent Variable: AA, ω-6/ DHA, ω-3 in LIMA  Independent variables: HTN – hypertension, HL- hyperlipidemia, FH- Family history of CAD, MI- Prior myocardial infarction, Cho- Cholesterol, LDL- Low density lipoprotein, HDL- High density lipoprotein, Tri- Triglyceride, DM- Diabetic Mellitus | | | | | | | | |

**S4 Table I. Linear regression of AA, ω-6/ DHA, ω-3 in RA with confounding variables**

| **Coefficients^a^** | | | | | | | | |
| --- | --- | --- | --- | --- | --- | --- | --- | --- |
| Model | | Unstandardized Coefficients | | Standardized Coefficients | t | Sig. | 95.0% Confidence Interval for B | |
|  |  | B | Std. Error | Beta |  |  | Lower Bound | Upper Bound |
| 1 | (Constant) | 1.170 | 8.182 |  | .143 | .889 | -17.061 | 19.401 |
|  | Age | .022 | .060 | .105 | .357 | .729 | -.113 | .156 |
|  | Gender | -2.920 | 1.791 | -.636 | -1.630 | .134 | -6.910 | 1.071 |
|  | HTN | -1.627 | 1.429 | -.386 | -1.139 | .281 | -4.812 | 1.557 |
|  | HL | 1.803 | 1.161 | .427 | 1.553 | .151 | -.783 | 4.390 |
|  | FH | 1.692 | .998 | .482 | 1.697 | .121 | -.530 | 3.915 |
|  | Smoking | 1.253 | 1.337 | .331 | .937 | .371 | -1.727 | 4.233 |
|  | MI | .668 | 1.859 | .169 | .359 | .727 | -3.475 | 4.811 |
|  | Cho | -.046 | .027 | -.700 | -1.696 | .121 | -.106 | .014 |
|  | LDL | .062 | .040 | .744 | 1.534 | .156 | -.028 | .152 |
|  | HDL | .080 | .074 | .345 | 1.090 | .301 | -.084 | .245 |
|  | Tri | .004 | .008 | .184 | .477 | .643 | -.014 | .022 |
|  | DM | -.405 | 1.373 | -.116 | -.295 | .774 | -3.463 | 2.654 |
| a. Dependent Variable: AA, ω-6/ DHA, ω-3 in LIMA  Independent variables: HTN – hypertension, HL- hyperlipidemia, FH- Family history of CAD, MI- Prior myocardial infarction, Cho- Cholesterol, LDL- Low density lipoprotein, HDL- High density lipoprotein, Tri- Triglyceride, DM- Diabetic Mellitus | | | | | | | | |
